# Relationships Between Extreme Ambient Temperatures, Neighborhood Structural Deprivation, and Fatal Police Violence: A Case-Crossover Analysis

**DOI:** 10.1007/s11524-026-01079-x

**Published:** 2026-05-03

**Authors:** Janelle R. Edwards, Gabriel L. Schwartz, Mark A. Hernandez, Sharrelle Barber, Shreya Patel, Jaquelyn L. Jahn

**Affiliations:** 1 The Ubuntu Center On Racism, Global Movements, and Population Health Equity, Dornsife School of Public Health, Drexel University, Philadelphia, PA USA; 2 Urban Health Collaborative, Dornsife School of Public Health, Drexel University, Philadelphia, PA USA; 3 Department of Health Management and Policy, Dornsife School of Public Health, Drexel University, Philadelphia, PA USA; 4 Department of Epidemiology and Biostatistics, Dornsife School of Public Health, Drexel University, Philadelphia, PA USA

**Keywords:** Police violence, Temperature, Neighborhood deprivation, Climate, Urban health, Policy

## Abstract

Police violence is increasingly recognized as a public health crisis, disproportionately affecting Black, Indigenous, and other communities of color due to long-standing patterns of racialized surveillance and disinvestment. Environmental stressors such as heat have also been linked to increased aggression, stress reactivity, and violence, suggesting that as climate change drives more frequent and intense extremes in temperature, these conditions may amplify existing risks of fatal police encounters. This study evaluated whether extreme ambient temperatures were associated with fatal police violence and whether structural neighborhood deprivation modified this relationship. Our nationwide case-crossover analysis examined daily maximum temperature and fatal police violence in the United States (2013–2024) using data from Mapping Police Violence. We estimated odds ratios across percentiles of the temperature distribution and analyses were stratified by neighborhood-level measures of deprivation, using Index of Concentration at the Extremes metrics for education, income, racialized income, and homeownership. Our main analysis revealed that compared to the median temperature (23.5 °C), the odds of fatal police violence at the 5th temperature percentile were reduced by 12% (95 percent CI: 0.806 to 0.955), while the odds at the 99th percentile were increased by 11% (CI: 1.037 to 1.185). While there was limited evidence of effect modification by neighborhood deprivation metrics, we found neighborhoods with higher levels of deprivation were disproportionately burdened by fatal police violence. These findings highlight the importance of temperature as a determinant of fatal police violence, suggesting that policies that address neighborhood deprivation and fatal policing may be needed on a warming planet.

**Supplementary Information:**

The online version contains supplementary material available at 10.1007/s11524-026-01079-x.

## Introduction

Police violence has increasingly been recognized as a public health crisis, highlighting how systemic patterns of harm disproportionately impact Black, Indigenous, and other communities of color [1–3]. While academic attention to these issues has grown in recent years, activists and community members have long documented and resisted structural racism and its role in fatal police violence [4–7]. Recent scholarship also recognizes that racial inequities in fatal police violence result from longstanding patterns of police surveillance and disinvestment in segregated communities [1–7]. These frameworks that draw attention to the structural determinants of fatal police violence provide important context for examining how environmental factors may intersect with and exacerbate risks of fatal police violence [8].

One such environmental factor that has been understudied in research on fatal police violence is climate—in particular, ambient temperature. Structurally disinvested neighborhoods, often characterized by limited green space, inadequate housing infrastructure, and poor access to cooling resources, contribute to localized heat island effects, particularly in urban settings [9]. These and related structural factors are also determinants of violence, which has been shown to worsen during periods of extreme heat [10–13]. However, there is limited research on weather extremes and fatal police violence specifically [14, 15]. Even less is known about the impact of extreme cold, highlighting a critical gap in the literature. This gap is especially pressing as climate change, driven by rising greenhouse gas emissions, is making extreme temperatures, both hot and cold, increasingly frequent and intense, heightening the risk of temperature-related health and safety threats and emphasizing the need to better understand their potential role in fatal police violence.

**Fig. 1:**
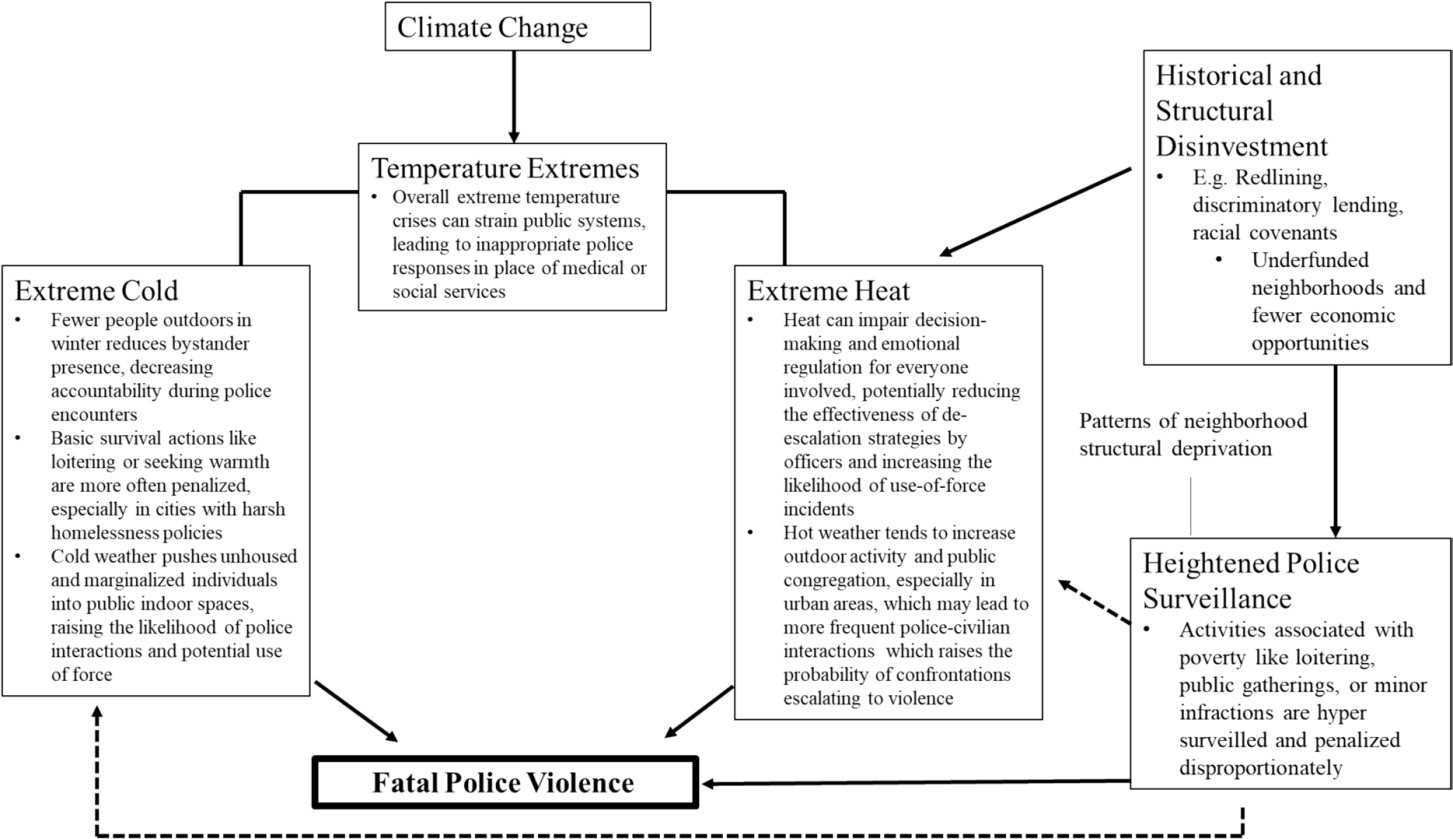
Conceptual framework illustrating the potential pathways linking extreme temperatures to fatal police violence

Moreover, there is limited examination of how racialized structural deprivation intersects with both temperature and fatal police violence. Our conceptual framework (Fig. 1) incorporates prior research on place-based climate vulnerability and neighborhood-level exposure to policing and outlines pathways through which spatial deprivation and environmental conditions may jointly shape the risk of fatal police violence. Historical racialized disinvestment has cemented present-day structural neighborhood deprivation and police surveillance, which may potentially compound the effects of extreme temperature on the risk of police violence. Briefly, the hypothesized pathways linking extreme hot temperatures to fatal police violence include increased officer aggression under heat stress, more frequent civilian–police interactions due to outdoor congregation, impaired de-escalation capacity, and the overlap of intense heat exposure and increased surveillance. In cold weather, pathways may include the criminalization of survival behaviors (e.g., seeking warmth), increased indoor surveillance and targeting of unhoused individuals, reduced bystander scrutiny of police actions, strain on emergency systems resulting in inappropriate police dispatch, and intensified surveillance in under-resourced communities. Collectively, these pathways highlight the need to examine how climate stressors interact with structural inequities to shape patterns of police violence in marginalized communities.

The objective of this study is therefore to examine the associations between extreme ambient temperatures, both hot and cold, and fatal police violence in the United States. Given prior research on the role of place-based racial inequities as determinants of both fatal police violence and temperature vulnerability, we further aim to assess how these associations may be modified by neighborhood-level indicators of structural deprivation, such as poverty, housing instability, educational attainment, and racialized income segregation. We hypothesize that (1) extreme temperatures will be associated with increased odds of fatal police violence, and (2) the magnitude of these associations will be heightened in neighborhoods that experience greater structural deprivation compared to more resourced communities.

## Methods

We conducted a time-stratified case-crossover analysis of associations between ambient daily temperature and fatal police violence across the contiguous United States.

### Analytical Variables

#### Case: Fatal Police Violence

Fatal police violence was defined as any encounter with law enforcement including shootings, physical assaults, or vehicular incidents that resulted in death, either on the day of the encounter or from related injuries. We obtained data from the Mapping Police Violence (MPV) project [16], a community science initiative that aggregates incidents using a structured multi-source process. Reports were identified through media searches, extracted and verified by two independent researchers, and geocoded with point locations. MPV excludes deaths not directly caused by police action, such as suicides or unrelated car crashes. We analyzed fatal police violence recorded from January 1, 2013, through December 31, 2024. These data are necessary because (1) no comprehensive Federal database exists [17], (2) Federal data suffers from extensive underreporting, and (3) capture-recapture analyses show that MPV’s methods provide the most complete data currently available [18].

The data included individual-level information on victims, including the geographic coordinates of the encounter, date, age, gender, race, and the cause of death. 

#### Exposure: Meteorological Data

Meteorological data were obtained from the Parameter elevation Regressions on Independent Slopes Model (PRISM) developed by Oregon State University [19]. We downloaded daily 4 km grids within which maximum temperature, precipitation, and mean temperature were calculated, from 2013 to 2024. We selected daily maximum temperature (DT_max_) as our primary variable, aligning with prior studies linking extreme heat to adverse outcomes [12, 20, 21]. Meteorological data were linked by date and location to fatal police violence and spatially joined to census tracts for stratified analyses.

#### Modifiers: Neighborhood Indicators of Structural Deprivation

We utilized 2017–2021 American Community Survey (ACS) 5-year estimates to derive several census tract measures of the Index of Concentration at the Extremes (ICE), a metric designed to quantify spatial social polarization. The ICE is calculated using the following formula:

$$ICE=\left(A_i-P_i\right)/T_i$$

where A_i_, P_i_, and T_i_ respectively correspond to the number of persons categorized, in the* i*th census tract, as belonging to: the most privileged extreme, the most deprived extreme, and the total population whose privilege level was measured. The ICE ranges from −1 to 1, where −1 and 1 indicate that 100% of the population is concentrated into the most extreme groups for deprivation and for privilege, respectively [22].

We calculated ICE for several dimensions of structural neighborhood privilege and deprivation relative to the tracts across the entire country. For income, the privileged group included individuals in the top 20th percentile of household income, while the deprived group included those in the bottom 20th percentile. We also constructed a combined measure of ICE by income and race/ethnicity, where the privileged group consisted of non-Hispanic White individuals in the top 20th income percentile and the deprived group consisted of non-Hispanic Black in the bottom 20th income percentile. ICE for homeownership was based on individuals residing in owner-occupied (privileged) versus renter-occupied (deprived) housing units. Finally, ICE for educational attainment was defined using adults aged 25 and older, with the privileged group having at least a four-year college degree and the deprived group lacking a high school diploma.

### Statistical Analysis

In this study, we employed a time-stratified case-crossover design, a method used for examining acute outcomes and transient exposures such as weather variables. This approach allows each participant to serve as their own control, effectively accounting for time-invariant individual-level confounders such as age, gender, and location without the need for explicit adjustment [23]. No cases are excluded or removed in this method. We used conditional logistic regressions to calculate Odds Ratios (OR) and associated 95% Confidence Intervals (CIs) that estimated the relationship between DT_max_ and fatal police violence. Cases (fatal police violence deaths) were matched to control days occurring in the same location, on the same day of the week, and within the same month and year using a time-stratified case-crossover design. All eligible control days meeting these criteria were included, with the case day itself excluded from the control set. For example, if a fatal police encounter occurred on Thursday, July 6, 2023, the matched control days would be Thursday, July 13; Thursday, July 20; and Thursday, July 27, 2023. This matching strategy allowed us to assess whether temperature on the case day differed meaningfully from temperatures on comparable control days. We identified the optimal parameterization for DT_max_ across the study period based on minimization of the AIC statistic, comparing different models with different parameterizations (linear term, or natural cubic spline term with varying degrees of freedom for the spline term, ranging from 2 to 4). We found that the ideal parametrization for DT_max_ was to model it linearly.

We adjusted for US major holidays to account for their potential confounding effect on the association between temperature and fatal police violence. Holidays can introduce short-term shifts in social behavior (e.g., increased alcohol consumption), police practices (e.g., heightened deployment), and population mobility (e.g., larger gatherings), which may coincide with temperature fluctuations, particularly because many holidays cluster in certain seasons (e.g., summer). Because this study uses a case-crossover design, each individual serves as their own control, inherently controlling for all time-invariant individual-level characteristics. As a result, factors such as alcohol use or weapons carrying, which are not expected to vary between case and control periods, cannot be estimated as covariates in this study design. Accordingly, we did not adjust for these factors in the models.

We produced extracted OR estimates at 5th (2.5 °C), 10th (7.0 °C), 25th (15.0 °C), 50th (23.5 °C), 75th (29.6 °C), 90th (33.5 °C), 95th (35.4 °C) and 99th (40.0°C) percentiles for the DT_max_ distribution and plotted the relationship between fatal police violence and DT_max_ with the median (50th percentile) daily temperature value (23.5 °C) as a referent.

We estimated stratified models across neighborhood census tract variables based on a priori hypotheses that they indicate neighborhood deprivation and the presence of hypersurveillance and therefore might modify associations between fatal police violence and ambient temperatures. Stratification was performed based on the ICE for education deprivation, homeownership deprivation, racialized income deprivation, and overall income deprivation. Each variable was categorized into terciles. Terciles were defined using the national census tract-level distribution of ICE values and assigned to each case based on the census tract where the fatal police encounter occurred (with highest levels of deprivation in Tercile 1 and lowest levels in Tercile 3). We also calculated case counts and incidence rates within each tercile. Incidence rates were computed by dividing the number of cases in each census tract by the total population of that tract, using population estimates from the ACS (2017–2021). Associations between temperature and odds of fatal police violence across stratified models appeared linear at the extreme ends of the temperature distribution based on visual inspection; therefore, to make comparisons across terciles of each neighborhood deprivation variable, we reported stratified estimates at the 5th and 99th percentiles of DT_max_. Lastly, we conducted the Cochran's Q test and reported the associated p-value to statistically assess heterogeneity of associations across terciles of each ICE modifier [24].

### Sensitivity Analyses

We conducted a series of sensitivity analyses. Firstly, we conducted a stratified analysis by geographic region to account for regional heterogeneity in both exposure and outcome patterns, as temperature distributions, policing practices, and population demographics can vary significantly across regions. Secondly, we re-ran our main analyses with additional adjustments for time-varying meteorological factors, including precipitation. Precipitation was adjusted for by adding a categorical term to the model based on the distribution of rainfall during the study period: (0: = 0 mm of rainfall, 1: = < than the mean of 2.68 mm, and 2: = > than 2.68 mm of rainfall). Lastly, because DT_max_ reflects daily extremes and does not capture the overall thermal burden, we re-ran the main unstratified analyses using daily mean temperature DT_mean_ instead of DT_max_. DT_mean_ was modeled linearly, based on the minimization of the AIC statistic (tested with df 1–4), with 17.1 °C as the reference value in the sensitivity analysis.

All statistical analysis was conducted using R studio version 3.4.1. All data are publicly available, and IRB approval was not required.

## Results

### Descriptives

Table 1 presents individual- and census tract-level characteristics of victims in fatal police violence from 2013 to 2024 (*n* = 13,972). Most victims were male (93.2%), aged 36–55 (38.5%), and non-Hispanic White (45.2%). The majority of deaths resulted from gunshot wounds (92.9%). Regionally, victims were concentrated in the South (42.9%), followed by the West (33.0%), Midwest (16.4%) and Northeast (7.7%). Fatalities were distributed evenly across seasons: summer (26.1%), fall (23.7%), winter (24.4%), and spring (25.8%).

**Table 1:** Descriptives of victims of fatal police violence (*n* = 13,972) between 2013 and 2024 across the contiguous United States

		Number (%)
Gender		
	Female	786 (5.6%)
	Male	13,154 (93.2%)
	Non-Binary	4 (0.001%)
	Transgender Female	3 (0.001%)
	Transgender Male	3 (0.001%)
	Unknown	22 (0.2%)
Age		
	<18	296 (2.2%)
	18–25	2297 (17.1%)
	26–35	4281 (31.8%)
	36–55	5186 (38.5%)
	56 and more	1403 (10.4%)
Race		
	Non-Hispanic Black	3597 (25.7%)
	Non-Hispanic White	6313 (45.2%)
	Hispanic	2511 (18.0%)
	Asian	214 (1.4%)
	Native Hawaiian and Pacific Islander	85 (0.6%)
	Native American	201 (1.4%)
	Unknown	1051 (7.5%)
Primary cause of death		
	Gunshot	12,978 (92.9%)
	Beaten/Baton/Bean bag	51 (0.37%)
	Asphyxiated/Strangulation	10 (0.07%)
	Physical restraint	102 (0.73%)
	Pepper Spray/Taser	373 (2.7%)
	Vehicular/PIT Stop/car crash	403 (2.9%)
	Other	29 (0.21%)
	Unclear	18 (0.13%)
Geographic region^1^		
	Northeast	1,077 (7.7%)
	Midwest	2,281 (16.4%)
	South	5,974 (42.9%)
	West	4,594 (33.0%)
Season		
	Summer	3,615 (26.1%)
	Fall	3,295 (23.7%)
	Winter	3,387 (24.4%)
	Spring	3,573 (25.8%)

^1^Based on United States (U.S) Census geographic regions

Supplemental Table 1 presents descriptives for neighborhood deprivation variables across the study period, from 2013 to 2024. Across all four ICE metrics, incidence rates and counts of fatal police violence were highest in the most deprived (tercile 1) and consistently decline across terciles 2 and 3. ICE Income showed the steepest drop, from 6.022 per 100,000 in tercile 1 to 4.685 per 100,000 in tercile 3, while ICE Education had the smallest change, from 5.661 per 100,000 to 5.058 per 100,000 across those same terciles. Both ICE Residential Income and ICE Homeownership followed a similar downward pattern.

### Associations Between Daily Maximum Temperature (DT_max_) and Fatal Police Violence

**Fig. 2:**
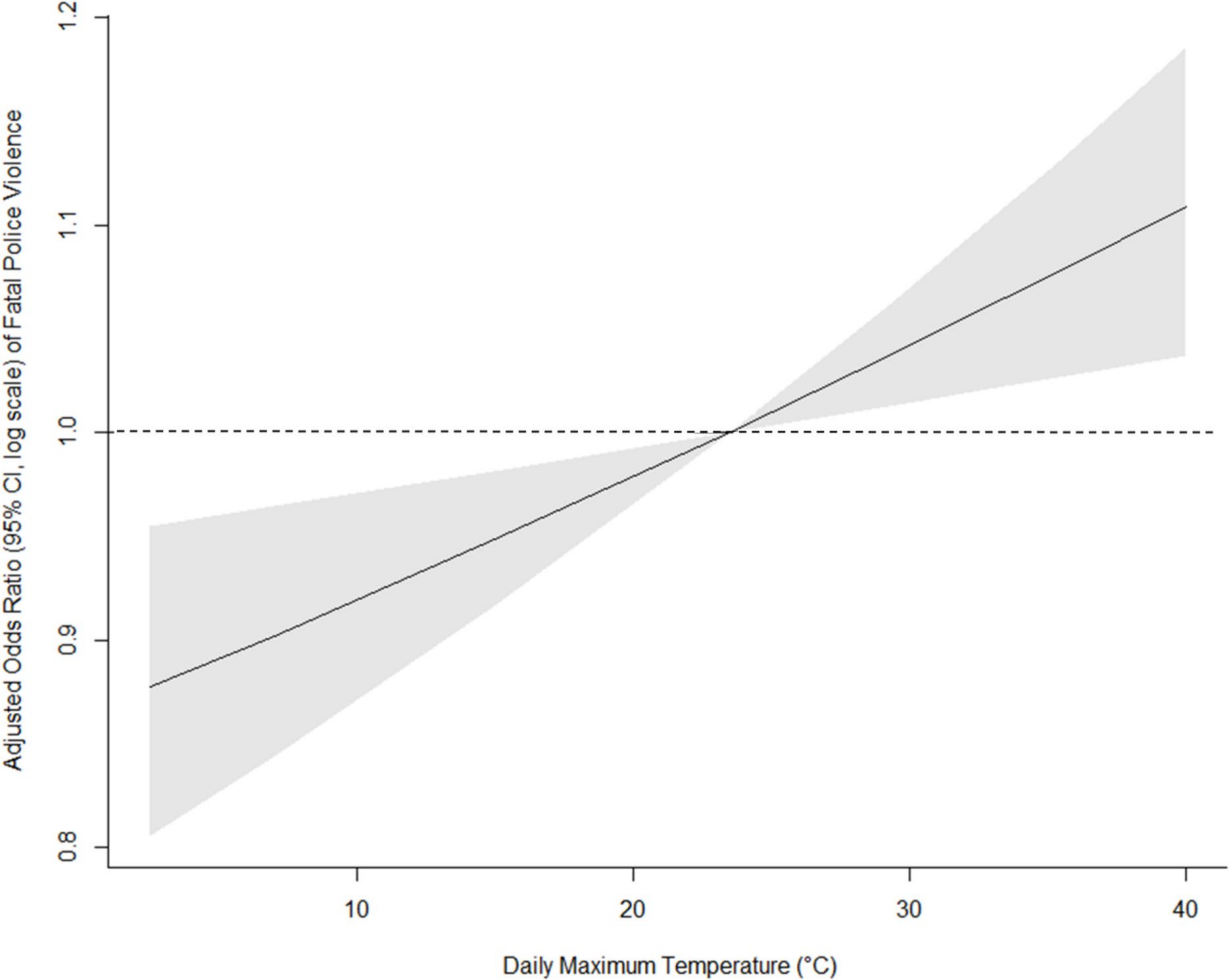
Plotted odds ratios (95% confidence intervals shaded grey) estimates of association between DT_max_ and fatal police violence between years 2013 and 2024. Odds ratios were estimated from conditional logistic regression where DT_max_ was modeled linearly and 23.5 °C was used as the referent to show how increasingly higher or increasingly lower temperatures than the median temperature across the study period affected the odds of fatal police violence. Model was adjusted for United Status major holidays. The dotted horizontal line corresponding to 1 on the y axis represents the null

Figure 2 presents results from models with adjustment for holidays, showing that colder temperatures (below the median) were protective, while odds increased with hotter temperatures. The odds of fatal police violence increased progressively as temperatures rose above the referent of 23.5 °C and decreased as temperatures fell below this threshold.

Supplemental Table 2 details the relationship between the extracted odds ratios of fatal police violence and DT_max_, with adjustment for holidays, which showed similar patterns. Using the median DT_max_ (23.5 °C) as the reference, the odds of a fatal police encounter at the 5th percentile of DT_max_ were reduced by 12% (95% CI: 0.806–0.955), while the odds at the 99th percentile were significantly increased by 11% (95% CI: 1.037–1.185).

### Stratifications by Indicators of Neighborhood Deprivation

**Fig. 3:**
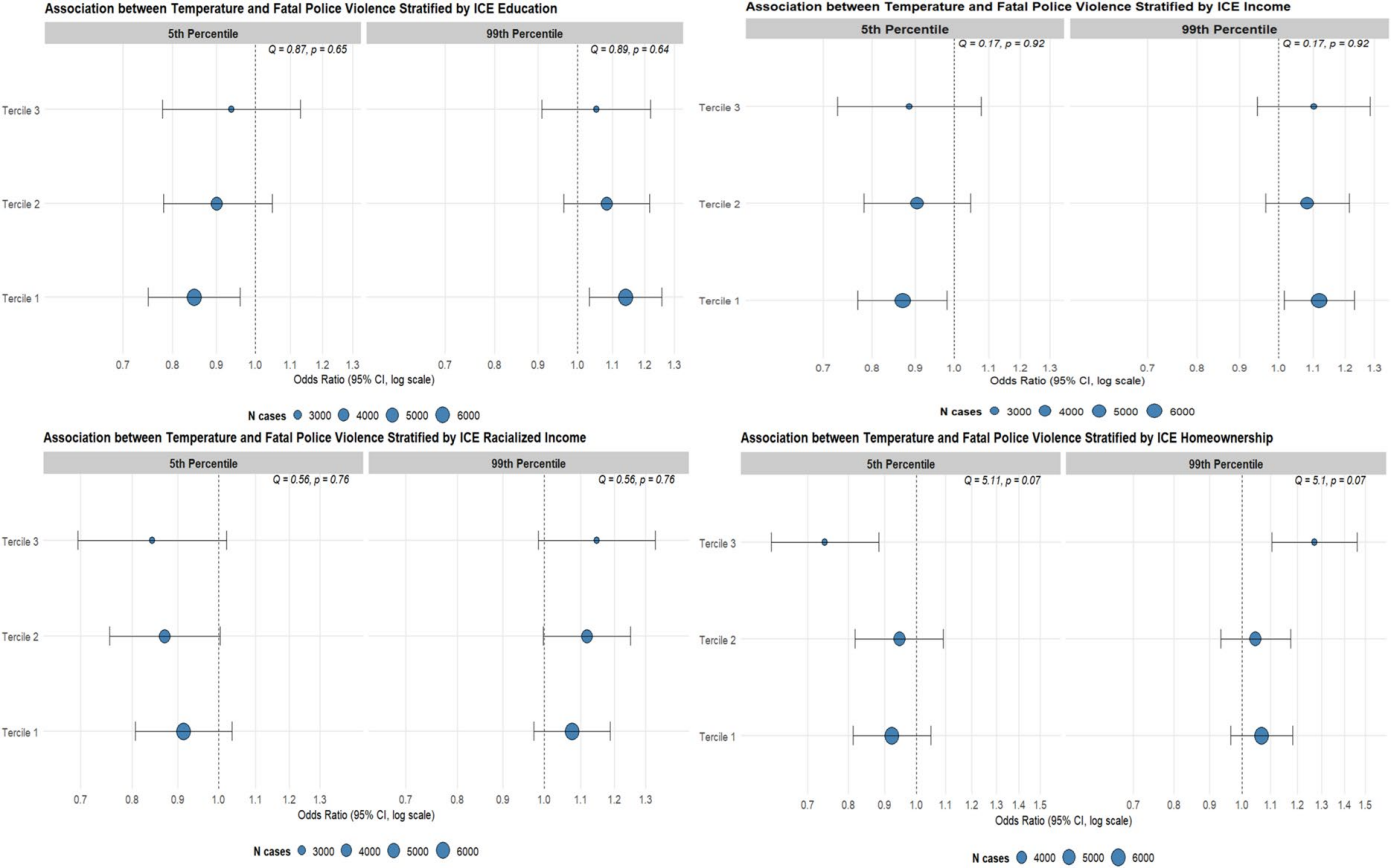
Odds ratios and associated confidence intervals for holiday adjusted associations between fatal police violence and extreme cold (5th percentile: 2.5 °C) and hot (99th percentile: 40 °C) temperatures, based on DT_max_ percentiles from 2013 to 2024, using 23.5 °C as the referent, stratified by various ICE metrics of neighborhood-level deprivation. Tercile 1 indicates that neighborhoods were most deprived, while Tercile 3 indicates that neighborhoods were most privileged. The size of each plotted symbol represents the number of cases of fatal police violence within each stratified group

Figure 3 shows the odds ratios and associated confidence intervals for holiday-adjusted associations between fatal police violence and extreme cold and hot temperatures, based on DT_max_ percentiles stratified by various ICE metrics of neighborhood-level deprivation. Terciles with the highest levels of deprivation for income and education displayed modestly higher odds ratios than more advantaged terciles, particularly during intense heat. However, that pattern was reversed for racialized income and homeownership, with higher odds ratios in less disadvantaged areas. Moreover, comparing terciles within each ICE measure, confidence intervals were wide, and Q tests for heterogeneity were non-significant in all cases (all *p* > 0.05), indicating no evidence of effect modification.

### Sensitivity Analyses

Supplemental Table 3 displays adjusted odds ratios for fatal police violence across percentiles of DT_max_ stratified by geographic region, using the 50th percentile (23.5 °C) as the reference. Higher temperatures were significantly associated with increased odds of fatal police violence in the South, with elevated risks observed from the 75th to the 99th temperature percentiles. This pattern was also observed in the West, though confidence intervals were wider and somewhat attenuated. In contrast, estimates were much smaller and statistically insignificant in the Northeast and essentially null in the Midwest. Further, we adjusted our main models for U.S. holiday and precipitation and found results were similar to main analysis (Supplemental Table 4). Lastly, in contrast to the main analysis, which used DT_max_ and showed statistically significant associations at both cold and hot extremes, associations with DT_mean_ were generally weaker and not statistically significant (Supplemental Table 5).

## Discussion

### Summary of Findings and Relation to Research

We conducted a nationwide case-crossover study to assess how extreme ambient temperatures, both hot and cold, affected the odds of fatal police violence. As hypothesized, extreme heat was associated with progressively higher odds of fatal police violence. Our results align with prior research [15] that examined county-level and monthly variations in temperature and their effects on civilian deaths involving conducted electrical weapons (e.g., Tasers) and physical restraints by police. The researchers found that each additional hot day (above 32 °C) was associated with a 5.3% increase in monthly civilian deaths compared to days within the 12 °C–17 °C range. Similarly, Martinson et al. [14] investigated the relationship between outdoor temperature and fatal police shootings and reported that a 5 °C increase in same-day maximum temperature was associated with a 3.3% increase in the odds of fatal police shootings (95% CI = 1.002, 1.065).

Contrary to our initial hypothesis that both temperature extremes would increase the odds of fatal police violence, we found that extreme cold was associated with a modest reduction in the odds of fatal police violence compared to the referent temperature. To our knowledge, no prior studies have explicitly examined the relationship between cold temperatures and fatal police violence. Colder temperatures may be protective by reducing outdoor activity and social interactions, compared to moderate temperatures, thereby limiting opportunities for civilians and police to interact.

In this analysis, we also examined whether neighborhood-level indicators of structural deprivation modified the association between extreme temperatures and fatal police violence. We hypothesized that the association between temperature and fatal police violence would be greater in more deprived neighborhoods. Our descriptive analysis revealed higher incidence rates of fatal police violence in areas with greater structural deprivation. Although statistically significant associations were observed in the most deprived neighborhoods for income and education, substantial overlap in confidence intervals across ICE levels suggests limited evidence of effect modification. Additionally, stratified models and formal tests of heterogeneity did not show statistically significant effect modification. However, even in the absence of strong relative effect modification, the higher baseline burden of police violence in structurally deprived neighborhoods means that increasing instances of extreme heat could result in larger absolute increases in these communities. Moreover, geographically stratified analyses further indicated that associations between temperature and fatal police violence were significantly elevated in the Southern United States, a region marked by a long legacy of racialized structural oppression, white supremacy, and racial terror as well as frequent exposure to extreme heat.

### Strengths and Limitations

This study has limitations. First, our methods capture how temperature fluctuations in a neighborhood affect the risk of a fatal police violence occurring in that location, but they may not reflect where the individuals involved actually lived. Specifically, the site of the fatal encounter may not accurately reflect the victim’s residential neighborhood or the area where they spent most of their life. This distinction is important because a growing body of research demonstrates that residential environments strongly shape health outcomes through long-term exposure to structural conditions such as neighborhood disinvestment and access to resources [25–27]. By using the location of the fatal police encounter without linking it with decedents’ residential neighborhoods, we risk overlooking the lived experiences and cumulative effects of neighborhood-level factors such as historical and contemporary patterns of investment and neglect that shape the social and environmental context of individuals’ daily lives. Therefore, while we can identify where deaths occurred, we cannot fully assess the impact of fatal police violence on the communities where the victims resided.

Second, our analysis focuses exclusively on fatal police violence and does not capture non-fatal encounters, such as shootings or other uses of force that do not result in death. Although these outcomes are not confounders of the temperature–fatal police violence relationship, temperature-related increases in police aggression or civilian–police encounters may manifest in less severe but more frequent forms of violence. Future research should examine whether temperature influences the broader spectrum of police use-of-force outcomes, including non-fatal shootings and injuries.

This study also has important strengths. A major strength is the use of a rigorously cross-validated national dataset that systematically documents incidents of fatal police violence, allowing for robust linkage with meteorological data on temperatures. This dataset, while potentially an undercount of incidents of fatal police violence, enhances the reliability and completeness of outcome ascertainment across geographies and time. Additionally, the application of a time-stratified case-crossover design makes our analyses robust to confounding that does not vary at the day level within a given month and year. Lastly, rather than stratifying solely by individual-level racial or ethnic identity, we examined how the associations between temperature extremes and fatal police violence were modified by neighborhood-level indicators of structural racism and concentrated deprivation. This approach allows for a more nuanced and spatially contextualized analysis of racialized disparities by focusing on the organized, place-based mechanisms through which systemic racism manifests [27]. By operationalizing racialized deprivation at the neighborhood level, we could appropriately capture the structural determinants that contribute to racialized patterns of police violence, moving beyond individual-level proxies to illuminate the broader systems that shape vulnerability to police-inflicted harm.

### Public Health Policy Implications

Our results suggest that climate change may amplify fatal police violence, which is both a direct health harm [28] and a community-level stressor that contributes to racial health inequities through population-wide psychological duress [29–31]. Our study demonstrates that extreme heat is associated with higher odds of fatal police violence across the United States, especially in the South. We find that while communities with higher levels of economic deprivation experience higher rates of fatal police violence, the impact of extreme heat on fatal police violence is consistent regardless of local socioeconomic conditions, highlighting the need for broad, population-wide policy interventions. Nonetheless, interventions to mitigate temperature-related risks in policing may be especially critical in these areas given their higher base rates of fatal police violence (such that a multiplicative increase in those neighborhoods in fatal police violence risk would yield larger absolute consequences). Strategies such as non-police, unarmed crisis response teams; enhanced officer training on de-escalation during extreme heat; revised operational protocols for high-temperature days; and strengthened accountability measures [32] could reduce the likelihood of deadly violence during extreme hot weather. Furthermore, relying on law enforcement to staff cooling or warming centers, conduct wellness checks, or respond to mental health crises during climate emergencies (and in general) can unnecessarily increase civilian–police interactions during high-risk periods. More broadly, given the well-documented dangers associated with widespread police surveillance and intervention, particularly in Black and other marginalized communities [33, 34], shifting responsibilities away from policing and toward community-led, public health-centered infrastructure is crucial. Alternatives that prioritize care over control include unarmed mental health crisis response teams [35] and neighborhood cooling initiatives like tree planting, shade structures, reflective roofing, community cooling centers, and expanded access to home and school air conditioning [36]. Interventions that strengthen the social determinants of safety, such as increasing educational opportunities, raising wages, and expanding affordable housing while protecting communities from displacement and gentrification, can further reduce harmful police violence and build resilience by intervening on the underlying determinants of criminalized behaviors.

In total, our findings highlight the intersection of climate change, public safety, and structural inequities as an emerging public health concern. Addressing these interconnected challenges will require collaboration among public health practitioners, policymakers, and communities to promote safety, resilience, and health equity as climate change continues.

## Supplementary Information

Below is the link to the electronic supplementary material.

ESM 1 (DOCX 31.6 KB)

## Data Availability

The data that support the findings of this study are openly available at https://mappingpoliceviolence.org.
