# Supplementary material for: Relationships Between Extreme Ambient Temperatures, Neighborhood Structural Deprivation, and Fatal Police Violence: A Case-Crossover Analysis
Source: J Urban Health. 2026 May 3;103(2):233–43. doi: 10.1007/s11524-026-01079-x (PMC13235678; doi:10.1007/s11524-026-01079-x)
Supplement: Supplementary file 1 — (DOCX 31.6 KB) [file 11524_2026_1079_MOESM1_ESM.docx]

| **Supplemental Table 1.** Descriptive summary statistics and incidence rates of fatal police violence^1^ for neighborhood deprivation^2^ variables across study period 2013-2024 | | | | | |
| --- | --- | --- | --- | --- | --- |
| Neighborhood Deprivation Variables ^2^ |  | Level | Distribution of ICE Metric | Count of fatal police violence | Rate of fatal police violence^1^ per 100,000 population |
|  | ICE Education |  |  |  |  |
|  |  | Tercile 1 | –0.636 (Min) ≤ x < 0.262 | 6758 | 5.661 |
|  |  | Tercile 2 | 0.262 ≤ x < 0.429 | 4427 | 5.276 |
|  |  | Tercile 3 | 0.429 ≤ x ≤ 1.000 (Max) | 2830 | 5.058 |
|  | ICE Income |  |  |  |  |
|  |  | Tercile 1 | –1.000 (Min) ≤ x < –0.128 | 6760 | 6.022 |
|  |  | Tercile 2 | –0.128 ≤ x < 0.0647 | 4486 | 5.105 |
|  |  | Tercile 3 | 0.0647 ≤ x ≤ 1.000 (Max) | 2769 | 4.685 |
|  | ICE Racialized Income |  |  |  |  |
|  |  | Tercile 1 | –1.000 (Min) ≤ x < 0.0363 | 6666 | 5.771 |
|  |  | Tercile 2 | 0.0363 ≤ x < 0.162 | 4488 | 5.307 |
|  |  | Tercile 3 | 0.162 ≤ x ≤ 1.000 (Max) | 2861 | 4.836 |
|  | ICE Homeownership |  |  |  |  |
|  |  | Tercile 1 | –1.000 (Min) ≤ x < 0.151 | 6299 | 5.707 |
|  |  | Tercile 2 | 0.151 ≤ x < 0.586 | 4642 | 5.170 |
|  |  | Tercile 3 | 0.586 ≤ x ≤ 1.000 (Max) | 3074 | 5.204 |
| 1. Incident rates were calculated by dividing the number of cases in each census tract by the total population of that tract, using population data from the American Community Survey (2017–2021), for each respective ICE tercile. 2. Neighborhood deprivation was indicated by ICE where lower values indicate higher deprivation and higher values indicate lower deprivation. ICE indicators were derived using census tract level population, economic, demographic data from the American Community Survey for years 2017–2021 | | | | | |

| **Supplemental Table 2**. Adjusted^1^ Odds Ratio and 95% confidence interval estimates of association between fatal police violence and daily maximum temperatures between 2013-2024 | |
| --- | --- |
| Temperature Percentile (^o^C) | Adjusted Odds ratio (95% CI) |
| 5^th^ (2.5) | 0.877 (0.806-0.955) |
| 10^th^ (7.0) | 0.902 (0.844- 0.964) |
| 25^th^ (15.0) | 0.948 (0.917 - 0.981) |
| 50^th^ (23.5; referent) | 1 |
| 75^th^ (29.6) | **1.039 (1.014 - 1.065)** |
| 90^th^ (33.5) | **1.065 (1.022- 1.109)** |
| 95^th^ (35.4) | **1.077 (1.027- 1.131)** |
| 99^th^ (40) | **1.109 (1.037- 1.185)** |
| 1. Adjustment for holiday   **Bolded** values indicate significance at p <0.05 | |

| **Supplemental Table 3.** Stratified geographic regional analysis with adjusted^1^ Odds Ratio and 95% confidence interval estimates of association between fatal police violence and daily maximum temperatures between 2013-2024 | | | | |
| --- | --- | --- | --- | --- |
|  | Adjusted Odds ratio (95% CI) | | | |
| Temperature Percentile (^o^C) | Northeast | West | Midwest | South |
| 5^th^  (2.5) | 0.970 (0.735- 1.292) | 0.867 (0.738-1.017) | 1.007 (0.847-1.1981) | 0.800 (0.697- 0.917) |
| 10^th^ (7.0) | 0.980 (0.786-1.223) | 0.894 (0.788-1.014) | 1.006 (0.878-1.153) | 0.839 (0.753- 0.934) |
| 25^th^ (15.0) | 0.990 (0.883-1.109) | 0.944 (0.885-1.007) | 1.003 (0.935- 1.076) | 0.914 (0.865- 0.966) |
| 50^th^  (23.5; referent) | 1 | 1 | 1 | 1 |
| 75^th^ (29.6) | 1.007 (0.928-1.094) | 1.043 (0.995-1.093) | 0.998 (0.949-1.05) | **1.068 (1.026- 1.111)** |
| 90^th^ (33.5) | 1.012 (0.885-1.158) | 1.071 (0.992-1.156) | 0.996 (0.917- 1.083) | **1.113 (1.042- 1.188)** |
| 95^th^ (35.4) | 1.015 (0.864-1.191) | 1.085 (0.99-1.188) | 0.996 (0.902-1.099) | **1.136 (1.051- 1.228)** |
| 99^th^ (40) | 1.020 (0.817-1.274) | 1.119 (0.986-1.27) | 0.994(0.867- 1.14) | **1.193 (1.071-1.329)** |
| 1. Adjustment for holiday, **Bolded** values indicate significance at **p <0.05** | | | | |

| **Supplemental Table 4**. Sensitivity analysis with additional adjusted^1^ Odds Ratio and 95% confidence interval estimates of association between fatal police violence and daily maximum temperatures between 2013-2024 | |
| --- | --- |
| Temperature Percentile (^o^C) | Adjusted Odds ratio (95% CI) |
| 5^th^ (2.5) | 0.882 (0.810 - 0.960) |
| 10^th^ (7.0) | 0.906 (0.848 - 0.969) |
| 25^th^ (15.0) | 0.951 (0.918 - 0.984) |
| 50^th^ (23.5; referent) | 1 |
| 75^th^ (29.6) | **1.037 (1.012 - 1.063)** |
| 90^th^ (33.5) | **1.062 (1.019 - 1.106)** |
| 95^th^ (35.4) | **1.074 (1.023 - 1.127)** |
| 99^th^ (40) | **1.104 (1.032 - 1.180)** |
| 1. Adjustment for holiday and precipitation   **Bolded** values indicate significance at p <0.05 | |

| **Supplemental Table 5**. Sensitivity analysis with adjusted^1^ Odds Ratio and 95% confidence interval estimates of association between fatal police violence and daily mean temperatures between 2013-2024 | |
| --- | --- |
| Temperature Percentile (^o^C) | Adjusted Odds ratio (95% CI) |
| 5^th^ (-1.5) | 0.977 (0.896 - 1.066) |
| 10^th^ (2.3) | 0.982 (0.916 - 1.053) |
| 25^th^ (9.2) | 0.990 (0.954 - 1.028) |
| 50^th^ (17.1; referent) | 1 |
| 75^th^ (23.7) | 1.008 (0.978 - 1.040) |
| 90^th^ (27.5) | 1.013 (0.965 - 1.063) |
| 95^th^ (29.1) | 1.015 (0.960 - 1.073) |
| 99^th^ (32.6) | 1.019 (0.948 - 1.096) |
| 1. Adjustment for holiday | |
